# Rasch-Based Calibration of Step-Based Task Achievement in a Surgical Clerkship: A Pilot Study of the Step Ladder System

**DOI:** 10.7759/cureus.114305

**Published:** 2026-08-10

**Authors:** Rumiko Omatsu, Takehiko Hanaki, Hiroaki Komatsu, Yoshiyuki Fujiwara, Masaru Ueki

**Affiliations:** 1 Division of Medical Education, Tottori University, Faculty of Medicine, Yonago, JPN; 2 Division of Urology, Tottori University, Faculty of Medicine, Yonago, JPN; 3 Division of Gastrointestinal and Pediatric Surgery, Tottori University, Faculty of Medicine, Yonago, JPN; 4 Division of Obstetrics and Gynecology, Tottori University, Faculty of Medicine, Yonago, JPN

**Keywords:** clerkship, competency-based education, curriculum diagnostics, medical students, rasch calibration, rasch model, step ladder system

## Abstract

Background

Competency-based education requires intended competency levels to be aligned with learners’ observed performance in authentic clinical settings. The Step Ladder System (SLS) is a three-step framework designed to structure clinical task progression and supervision expectations during clerkships. However, the empirical alignment between predefined SLS step categories and observed task-achievement patterns has not been examined.

Aim

This study aimed to use Rasch calibration to examine the empirical alignment between predefined SLS step categories and observed task-achievement patterns in a pilot surgical clerkship dataset.

Methods

We conducted a secondary analysis of physician-confirmed binary task-achievement records from 24 sixth-year medical students during a four-week gastrointestinal and pediatric surgery clerkship using a 159-task SLS checklist. Task achievement was coded as 1 if achieved at least once and 0 if not achieved. Rasch-calibrated item difficulty (β), interpreted as observed achievement difficulty within the clerkship context, was estimated using a dichotomous Rasch model with conditional maximum likelihood estimation (eRm package (R version 4.5.2, R Foundation for Statistical Computing, Vienna, Austria)). Items with invariant response patterns, defined as all-0 or all-1 responses, were summarized descriptively by step and response pattern and excluded from β estimation. Stepwise differences in β were examined using Kruskal-Wallis tests with Bonferroni-adjusted Dunn post hoc comparisons. The association between β and achievement rate was assessed using Spearman’s ρ as a parameter-orientation check.

Results

Of the 159 SLS tasks analyzed (Step 1: 59; Step 2: 53; Step 3: 47), 36 showed invariant response patterns and were excluded from β estimation (Step 1: 2; Step 2: 14; Step 3: 20). Among these, 35 were all-0 items and one was an all-1 item; all invariant items in Steps 2 and 3 were all-0. β-based analyses included 122 basic/free item parameters under the sum-to-zero parameterization (Step 1: 56; Step 2: 39; Step 3: 27). Median β values (IQR) increased across steps: −0.55 (−1.06 to 0.46) for Step 1, 0.46 (0.07 to 1.76) for Step 2, and 0.97 (0.46 to 0.97) for Step 3. β differed significantly across steps (χ²(2) = 27.48, p < 0.001, ε² = 0.214). Post hoc comparisons showed that Step 1 had significantly lower observed achievement difficulty than Steps 2 and 3 (both p < 0.001), whereas Steps 2 and 3 did not differ significantly (p = 0.712). β was strongly and inversely associated with achievement rate (ρ = −0.992, p < 0.001), consistent with the expected orientation of the item-difficulty estimates.

Conclusions

Rasch calibration of SLS achievement-log data suggested that Step 1 tasks were empirically distinct from higher-step tasks in this pilot surgical clerkship dataset, whereas Step 2 and Step 3 tasks were not clearly separated among the analyzed items, raising an unresolved question of whether this reflects pilot- scale measurement limitations or structural overlap in upper-step task definitions. The concentration of all 0 invariant items in higher steps suggests that limited task exposure and sparse achievement patterns may have affected upper-step calibration. These findings support the feasibility of using Rasch-calibrated achievement logs as a curriculum diagnostic tool to identify areas where predefined step categories, task definitions, and clerkship opportunities may require refinement.

## Introduction

Competency-based education requires explicit developmental pathways that guide learners from observation to supervised participation and, ultimately, toward greater clinical autonomy [1, 2]. Entrustable professional activities (EPAs) operationalize this progression by linking competence judgments to observable workplace tasks [3]. At the program level, implementation frameworks for competency-based medical education further emphasize the need for explicit design and evaluation across training stages [4]. However, stage-based training frameworks are often implemented before their alignment with learners’ observed performance in practice has been empirically examined [5, 6]. The present study addresses this issue by examining whether the predefined steps of the Step Ladder System (SLS) [7, 8], a step-based clerkship framework, are empirically aligned with students’ observed task-achievement patterns during a surgical clerkship.

The SLS is a three-step clerkship framework designed to structure clinical task progression and supervision expectations [7]. In this framework, Step 1 includes basic observational or simple procedural tasks, Step 2 includes intermediate tasks typically requiring partial assistance, and Step 3 includes tasks expected to require greater autonomy. Although the SLS is not an EPA framework per se, it shares with EPA-based approaches an emphasis on observable clinical tasks, progressive participation, and supervision expectations. The SLS has been implemented in clinical education settings, including obstetrics and gynecology, with reported educational utility [7]. More recently, the SLS was applied in a surgical clerkship and was associated with improved medical students’ self-efficacy, suggesting its potential utility as a learner-facing scaffold in surgical education [9].

Faculty members assigned each task to one of the three SLS steps by consensus; they did not assign numerical difficulty scores. In the present study, task difficulty was not a faculty-rated attribute but a Rasch-estimated item location parameter derived from observed binary achievement patterns. Intended step assignment and observed task-achievement difficulty are therefore related but distinct constructs. In clerkships, task achievement is influenced not only by intrinsic task complexity but also by case mix, service workflow, supervision context, and opportunities for participation [10, 11]. Accordingly, predefined step categories may not fully correspond to observed task-achievement patterns in a given clerkship setting.

Rasch modeling provides a probabilistic measurement framework for estimating item difficulty and learner location on a common latent logit scale [12]. In practical terms, tasks that are achieved by fewer students receive higher β values, whereas tasks achieved by more students receive lower β values, after placing student achievement tendency and task difficulty on the same logit scale. Guidance on Rasch interpretation emphasizes that estimated item parameters should be understood within the assumptions and context of the fitted model [13]. In medical education, Rasch-family approaches have been applied to assessment contexts such as common-content assessment items and objective structured clinical examinations [14-16]. For clerkship achievement logs, this framework may enable preliminary calibration of heterogeneous binary task-achievement items beyond raw completion proportions while making model assumptions explicit [12, 13, 17]. Unlike simple achievement percentages, which describe only how frequently each task was completed, Rasch calibration places student achievement tendency and task difficulty on a common logit scale, thereby enabling task difficulties to be compared while accounting for differences in students’ overall tendency toward task achievement. In this context, Rasch-estimated item difficulty should be interpreted as observed achievement difficulty within a specific clerkship exposure structure, rather than as intrinsic task difficulty or definitive clinical competence [12, 13, 17]. In this study, the latent continuum was defined as students’ underlying tendency toward task achievement within the observed clerkship context. Accordingly, item location parameters were interpreted as the degree to which each task was difficult to achieve under the actual clinical exposure, supervision, and workflow conditions of the clerkship, rather than as intrinsic procedural difficulty or definitive clinical competence.

Accordingly, this pilot study examined whether predefined SLS step categories were empirically aligned with observed patterns of task achievement in a surgical clerkship, using Rasch calibration to estimate task difficulty from students' achievement records. We interpreted the resulting findings as preliminary achievement log calibration intended for educational and curriculum-level refinement, rather than as definitive validation of the SLS as a competency assessment scale.

## Materials and methods

We used a pilot quantitative design with convenience sampling and conducted a secondary analysis of routinely collected SLS achievement logs from the Division of Gastrointestinal and Pediatric Surgery, Tottori University Faculty of Medicine, Yonago, Japan. The objective was to examine the empirical alignment between predefined SLS step categories and observed task-achievement patterns in an undergraduate surgical clerkship context. The SLS task list and predefined step classifications (version 1.7) are available in the Figshare dataset [18]. 

Participant details 

In 2025, 24 sixth-year medical students (15 males, nine females) completed a four-week gastrointestinal and pediatric surgery clerkship and participated in SLS-based task tracking. SLS procedures were explained during group orientation on day 1. Participation in SLS tracking was voluntary and did not affect course grades.

For all tasks, supervising physicians evaluated student performance and confirmed achievement or non-achievement according to departmental SLS criteria. Students did not self-rate task achievement for the analytic outcome; achievement status was based on supervising physician confirmation recorded during routine clerkship supervision. The analytic dataset was constructed from these physician-confirmed records as a 24 × 159 dichotomous matrix, with task achievement coded as 1 if achieved at least once and 0 if not achieved.

This study was a secondary analysis of de-identified routine educational records and was conducted using an institutional opt-out procedure via public disclosure. The protocol was approved by the Tottori University Faculty of Medicine Ethics Committee (Approval No. 25A164) and conducted in accordance with the Declaration of Helsinki.

Rasch calibration and statistical analysis

A one-parameter dichotomous Rasch model was fitted to the 0/1 task-achievement data matrix using the RM function in the eRm package (version 1.0.10) with conditional maximum likelihood estimation and a sum-to-zero identification constraint (sum0 = TRUE) in R version 4.5.2 (R Foundation for Statistical Computing, Vienna, Austria). No response data were missing. Person location and item difficulty were placed on a common latent logit scale [12]. In this pilot analysis, the latent continuum was interpreted as an underlying tendency toward task achievement in the clerkship context, rather than as definitive clinical competence. Accordingly, β was interpreted as observed achievement difficulty within the clerkship exposure structure.

Items with no response variability, defined as all-0 or all-1 response patterns within the sample, were summarized descriptively by step and response pattern and excluded from β estimation because they do not provide within-sample information for estimating item location. Invariant items were not removed as part of scale refinement; rather, they were excluded only from β estimation. These items were retained in descriptive summaries because their concentration in upper steps was considered a curriculum-relevant finding. Thus, 123 of 159 items were retained as non-invariant calibration candidates (Step 1: 57; Step 2: 39; Step 3: 27). Under the sum-to-zero parameterization, 122 basic/free item parameters (Step 1: 56; Step 2: 39; Step 3: 27) were used for β-based summaries and inferential analyses. Higher β values indicate greater observed achievement difficulty on the latent logit scale.

Although achievement judgments were physician-confirmed, the dataset did not contain a sufficiently structured rater-by-student-by-task design to support stable many-facet Rasch modeling. Multiple supervisors contributed to routine clinical confirmation, and rater assignment was not experimentally balanced across students or tasks. Therefore, rater severity could not be estimated as a separate facet in this pilot analysis. This limitation was considered when interpreting item-difficulty estimates as preliminary calibration parameters rather than definitive item measures.

Item-level infit and outfit mean-square statistics were inspected descriptively to screen for gross model-data misfit. We did not use fit statistics for item deletion because the purpose of this pilot analysis was exploratory curriculum calibration rather than construction of a high-stakes psychometric scale. Given the small number of learners and the routine educational-record structure of the dataset, formal tests of local independence, reliability/separation, and unidimensionality were considered underpowered and were not used as exclusion criteria. Stepwise differences in β were summarized using mean, standard deviation, median, interquartile range, and range and were tested using the Kruskal-Wallis test. Effect size for the Kruskal-Wallis test was reported as epsilon-squared (ε²), interpreted using conventional benchmarks (negligible, weak, moderate, strong, very strong) [19]. When the omnibus test was significant, Bonferroni-adjusted Dunn rank-based pairwise comparisons were performed. Achievement rates, defined as the proportion of students achieving each task, were used descriptively. The association between β and achievement rate was evaluated using Spearman’s rank correlation as a parameter-orientation check rather than as independent evidence of construct validity. Distributions and associations were visualized using boxplots and scatterplots. Analyses were conducted in R version 4.5.2 using the eRm, dplyr, FSA, and ggplot2 packages.

## Results

Item screening and Rasch calibration

A total of 159 SLS tasks were included in the dataset: 59 Step 1 tasks, 53 Step 2 tasks, and 47 Step 3 tasks. Thirty-six items showed invariant response patterns and were excluded from Rasch calibration because β could not be estimated from within-sample response variation. These invariant items were distributed as follows: Step 1, 2 items; Step 2, 14 items; and Step 3, 20 items. Among the 36 invariant items, 35 were all-0 items, and one was an all-1 item. All invariant items in Steps 2 and 3 were all-0. The remaining 123 non-invariant items were eligible for calibration: Step 1, 57 items; Step 2, 39 items; and Step 3, 27 items. Item-difficulty summaries and inferential analyses were based on 122 basic/free item parameters under the sum-to-zero parameterization (Step 1: 56; Step 2: 39; Step 3: 27).

Distribution of observed achievement difficulty across steps

**Figure 1:**
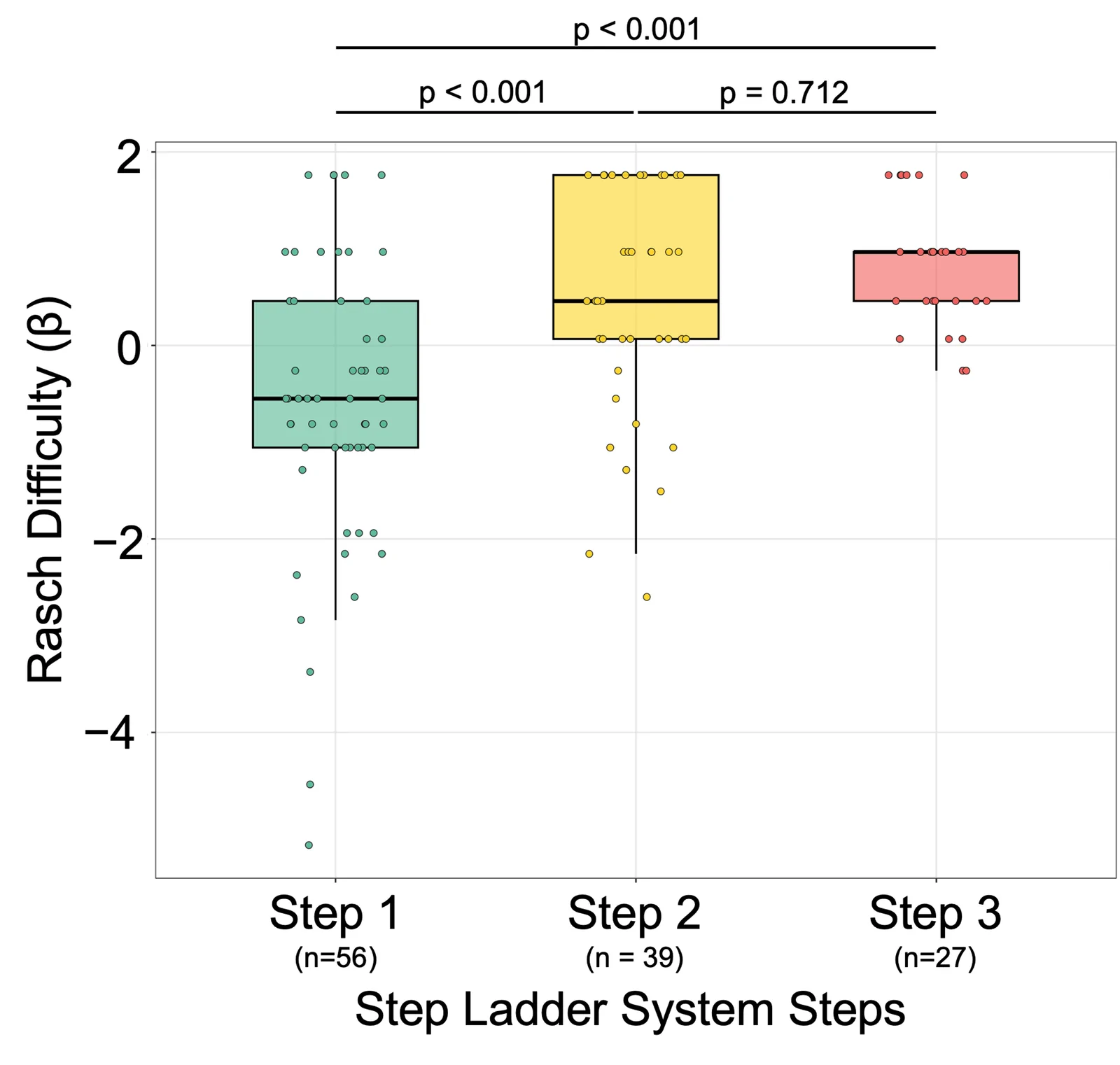
Distribution of Rasch-estimated item difficulty (β) across Step Ladder System (SLS) steps. Note: Box-and-whisker plots show the distribution of β values for Step 1, Step 2, and Step 3 tasks among the 122 items included in the analyses. Higher β values indicate greater observed achievement difficulty within the clerkship context. Step 1 showed lower median β values than Steps 2 and 3, whereas Steps 2 and 3 showed substantial overlap, consistent with the main findings. The figure was generated using ggplot2 in R version 4.5.2 (R Foundation for Statistical Computing, Vienna, Austria).

**Figure 2:**
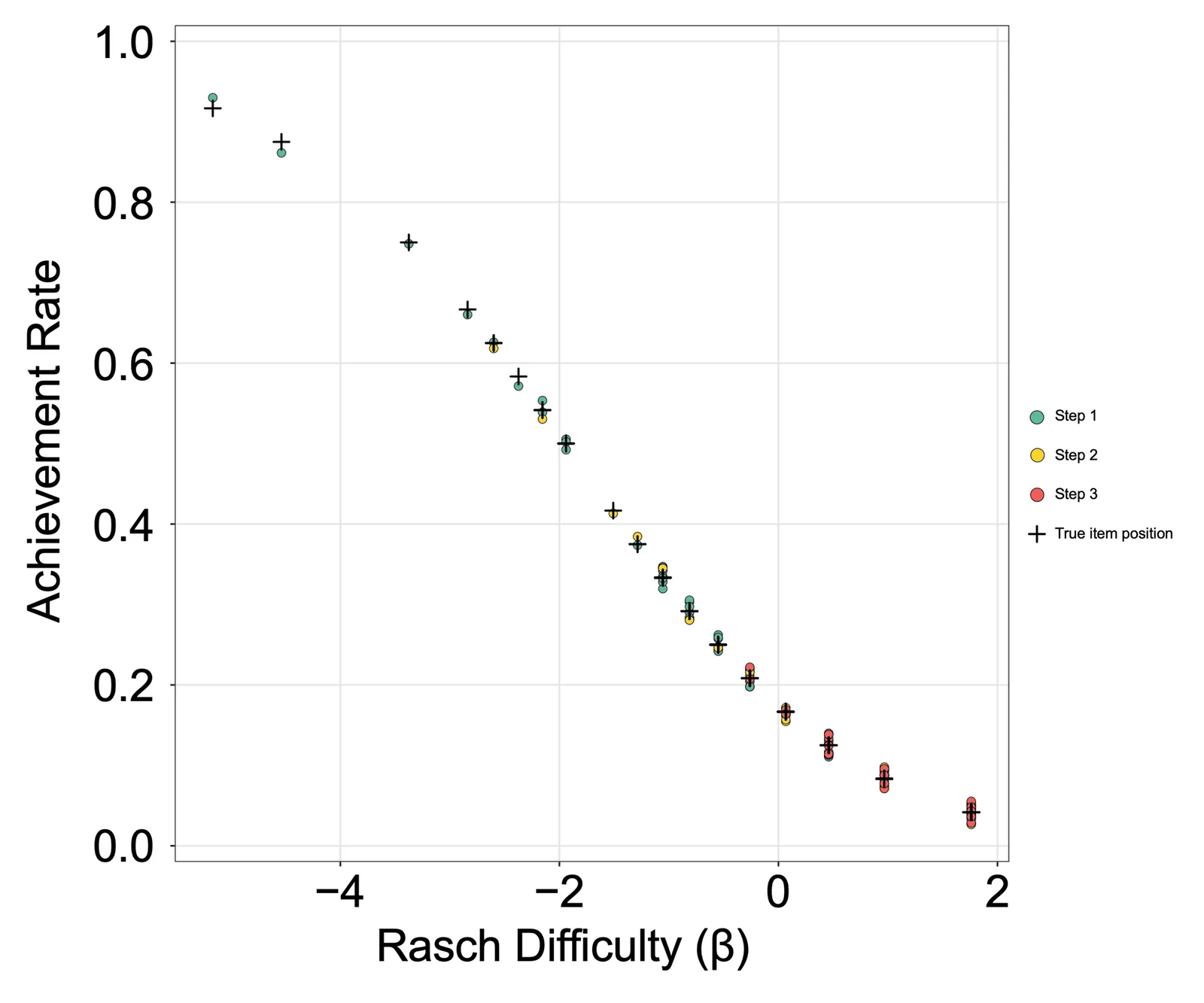
Relationship between Rasch-estimated item difficulty and achievement rate. Note: Scatterplot of Rasch-estimated item difficulty (β) versus task achievement rate, defined as the proportion of students achieving each task, among the same 122 items. To improve visibility when multiple items shared identical or near-identical coordinates, small vertical jitter was applied for display purposes only; the underlying values were unchanged. The strong inverse association between β and achievement rate (Spearman’s ρ = −0.992, p < 0.001) was reported as a parameter-orientation check, consistent with higher β values indicating greater observed achievement difficulty. The figure was generated using ggplot2 in R version 4.5.2 (R Foundation for Statistical Computing, Vienna, Austria).

The distribution of Rasch-estimated item difficulty, interpreted as observed achievement difficulty within the clerkship context, shifted upward with increasing SLS step level (Figure 1, Table 1). The association between achievement rate and β is summarized in Table 1 and visualized in Figure 2.

**Table 1:** Rasch calibration summary and stepwise statistical comparisons of observed achievement difficulty. Note: Stepwise Rasch-estimated item difficulty (β) summaries are based on 122 basic/free item parameters under the sum-to-zero parameterization. Invariant items were retained in dataset-level counts but excluded from Rasch calibration. Among the 36 invariant items, 35 were all-0 items, and one was an all-1 item; all invariant items in Steps 2 and 3 were all-0. Achievement rates were used descriptively and for the parameter-orientation check.

Measure		Step 1	Step 2	Step 3	Overall
Total tasks in dataset		59	53	47	159
Invariant items excluded from Rasch calibration (all-0 or all-1)		2	14	20	36
	all-0	1	14	20	35
	all-1	1	0	0	1
Non-invariant items (eligible for Rasch calibration)		57	39	27	123
Basic/free item parameters used for analysis		56	39	27	122
Mean β (SD)		−0.61 (1.46)	0.44 (1.18)	0.82 (0.63)	—
Median β (IQR)		−0.55 (−1.06 to 0.46)	0.46 (0.07 to 1.76)	0.97 (0.46 to 0.97)	—
Range of β		−5.16 to 1.76	−2.60 to 1.76	−0.26 to 1.76	−5.16 to 1.76
Kruskal–Wallis test comparing β across steps		—	—	—	χ^2^(2) = 27.48, p = 1.08×10⁻^6^, ε^2 ^= 0.214
Pairwise comparisons (Dunn, Bonferroni-adjusted)		—	—	—	1 vs 2: p < 0.001; 1 vs 3: p < 0.001; 2 vs 3: p=0.712
Correlation between β and achievement rate (Spearman)		—	—	—	ρ = −0.992 (p < 0.001)

Stepwise summaries of β were as follows: Step 1 (n = 56), mean β = −0.61 (SD 1.46), median β = −0.55 (IQR −1.06 to 0.46), and range −5.16 to 1.76; Step 2 (n = 39), mean β = 0.44 (SD 1.18), median β = 0.46 (IQR 0.07 to 1.76), and range −2.60 to 1.76; and Step 3 (n = 27), mean β = 0.82 (SD 0.63), median β = 0.97 (IQR 0.46 to 0.97), and range −0.26 to 1.76.

A Kruskal-Wallis test showed a significant difference in β across steps (χ²(2) = 27.48, p = 1.08 × 10⁻⁶, ε² = 0.214) corresponding to a moderate effect size according to conventional benchmarks for epsilon-squared [19]. Dunn’s post hoc test with Bonferroni adjustment showed significant differences between Step 1 and Step 2 (p < 0.001) and between Step 1 and Step 3 (p < 0.001), but not between Step 2 and Step 3 (p = 0.712). Thus, Step 1 showed significantly lower observed achievement difficulty than Steps 2 and 3, whereas Steps 2 and 3 were not clearly separated on the basis of their β distributions. Item-level infit and outfit mean-square statistics were inspected descriptively, but no items were excluded on the basis of fit statistics because no prespecified fit-based exclusion thresholds were applied in this exploratory pilot analysis.

Association between item difficulty and achievement rate

Across these 122 items, Rasch-estimated item difficulty was strongly and inversely associated with item-level achievement rate (Spearman’s ρ = −0.992, p < 0.001; Figure 2). This finding was consistent with the expected direction of the item-difficulty parameter and was interpreted as a parameter-orientation check rather than as independent evidence of construct validity.

## Discussion

This pilot Rasch analysis suggested partial empirical alignment between predefined SLS step categories and observed task-achievement patterns in a surgical clerkship. Step 1 tasks showed lower observed achievement difficulty than Step 2 and Step 3 tasks, supporting the intended distinction between lower-step and higher-step tasks in this dataset. However, Step 2 and Step 3 were not clearly separated among the analyzed items, indicating that the upper-step structure could not be stably distinguished under the current pilot conditions.

Two findings require particular attention. First, 36 of 159 tasks showed invariant response patterns and were excluded from primary Rasch calibration because they provided no within-sample response variation for item-location estimation [20]. These invariant items were concentrated in the higher steps (Step 2: n = 14; Step 3: n = 20), and all invariant items in Steps 2 and 3 were all-0. This indicates that a substantial subset of upper-step tasks did not contribute to within-sample calibration in this clerkship dataset [20, 21]. This finding should be interpreted not only as a technical limitation of Rasch estimation but also as a potentially important curriculum-level signal. The concentration of all-0 items in higher steps suggests that some upper-step tasks may not have been observable or achievable within the current four-week clerkship structure, potentially reflecting limited observation time, insufficient clinical opportunities, sparse student exposure, or tasks that were too advanced for routine undergraduate participation. Because these all-0 items were excluded from comparisons of item difficulty, the observed contrast between Steps 2 and 3 may have been attenuated.

Second, even among non-invariant items, Step 2 and Step 3 showed overlapping β distributions and a non-significant pairwise difference. This result should not be interpreted as definitive evidence against the conceptual hierarchy of the SLS; rather, it points to an unresolved question of whether the overlap primarily reflects insufficient measurement information under the current pilot conditions or a structural limitation in the upper-step definitions of the SLS.

Sparse achievement patterns, limited sample size, context-specific task exposure, and possible overlap in task definitions or behavioral anchors could all plausibly produce this pattern, and item calibration is known to become more stable with larger samples and more informative response distributions [22, 23]. Distinguishing between these explanations will require larger multi-cohort or multi-site data that account for task opportunity, clinical domain, rotation timing, and rater structure. If Step 2 and Step 3 become clearly separable once these factors are addressed, the present overlap would mainly reflect pilot-scale information limitations; persistent overlap would instead support conceptual refinement of the upper-step definitions.

These findings have practical implications for using SLS data as a curriculum diagnostic tool. Rather than treating the current analysis as definitive validation of the SLS as a competency assessment scale, Rasch-calibrated achievement logs may help identify where intended step progression and actual clerkship experience are misaligned. For example, Step 1 tasks with unexpectedly high β values may require clearer instruction or more reliable opportunities for participation. Step 2 or Step 3 tasks with invariant or sparse achievement patterns may require reconsideration of task definitions, expected achievement levels, or clerkship exposure design. Similarly, substantial overlap between Step 2 and Step 3 items may indicate the need for more explicit behavioral anchors distinguishing partial assistance from greater autonomy.

Several limitations should be considered. This was a single-institution pilot study with a small number of students, and the observed task-achievement patterns may have been influenced by local curricular organization, rotation structure, clinical case availability, and supervision practices, thereby limiting the generalizability of the findings to other institutions. Achievement judgments were based on routine workplace supervision, and rater-related variability, including differences in severity or leniency, could not be modeled explicitly because the dataset lacked a balanced rater-by-student-by-task structure. A many-facet Rasch model was therefore not applied. Infit and outfit statistics were inspected descriptively, but detailed fit diagnostics and threshold-based item decisions were beyond the scope of this pilot analysis. Formal tests of local independence, reliability/separation, and unidimensionality were not conducted as definitive psychometric diagnostics because of the small learner sample and the exploratory curriculum-calibration purpose of this study. Therefore, the present results should not be interpreted as evidence that the SLS task set constitutes a fully validated unidimensional measurement scale.

Future work should therefore move beyond simple replication with a larger sample and should be explicitly designed to test these competing explanations, prospectively capturing task-level opportunity, clinical domain, rotation timing, level of supervision, and rater information across a multi-cohort or multi-site dataset. This would allow sensitivity analyses excluding tasks without clinical opportunity, domain-stratified calibration, differential item functioning analyses across sites or clinical domains, and, where feasible, facet-explicit modeling of rater effects. With these extensions, SLS achievement-log calibration could be strengthened as a method for educational feedback, curriculum-level quality assurance, and iterative refinement of step-based clerkship frameworks.

## Conclusions

In this pilot Rasch calibration of SLS clerkship achievement logs, Step 1 tasks showed lower observed achievement difficulty than higher-step tasks, supporting the intended lower-versus-higher step distinction in this dataset. However, Step 2 and Step 3 tasks were not clearly separated among the analyzed items, with overlapping β distributions and non-significant pairwise differences. The concentration of all-0 invariant items in higher steps suggests that limited within-sample information, sparse achievement patterns, and context-specific task exposure may have affected upper-step calibration. Overall, these findings support the feasibility of using Rasch-calibrated SLS achievement logs as a curriculum diagnostic tool, while identifying priorities for refinement: clearer upper-step task definitions, larger multi-cohort or multi-site datasets, and opportunity-adjusted designs that can clarify whether the Step 2/3 overlap reflects pilot-scale measurement limitations or structural overlap in upper-step definitions.

## References

[1] Caretta-Weyer HA, Gisondi MA (2019). Design your clinical workplace to facilitate competency-based education. West J Emerg Med.

[2] Kar SS, Dube R, George BT, Bairy LK, Hajeer AH, Matalka II (2025). Implementation of United Arab Emirates competency framework for medical education in undergraduate medical curriculum. BMC Med Educ.

[3] ten Cate O (2017). Competency-based medical education and its competency frameworks. Competence-based Vocational and Professional Education.

[4] Van Melle E, Frank JR, Holmboe ES, Dagnone D, Stockley D, Sherbino J (2019). A core components framework for evaluating implementation of competency-based medical education programs. Acad Med.

[5] Alharbi NS (2024). Evaluating competency-based medical education: a systematized review of current practices. BMC Med Educ.

[6] Hawkins RE, Welcher CM, Holmboe ES, Kirk LM, Norcini JJ, Simons KB, Skochelak SE (2015). Implementation of competency-based medical education: are we addressing the concerns and challenges?. Med Educ.

[7] Komatsu H, Ueki M, Yamada N, Taniguchi F, Harada T (2025). Implementation and educational impact of the step ladder system: a structured clinical competency framework for obstetrics and gynecology. Int J Gynaecol Obstet.

[8] Komatsu H, Ueki M, Yamada N (2026). Educational implications of a tiered, competency-based “step ladder system”. J Obstet Gynaecol Res.

[9] Hanaki T, Omatsu R, Miyatani K, Kihara K, Komatsu H, Fujiwara Y, Ueki M (2026). Climbing the ladder of confidence: effects of the step ladder system on medical students' self-efficacy during a surgical clerkship. BMC Med Educ.

[10] Surmon L, Bialocerkowski A, Hu W (2016). Perceptions of preparedness for the first medical clerkship: a systematic review and synthesis. BMC Med Educ.

[11] Bosch J, Maaz A, Hitzblech T, Holzhausen Y, Peters H (2017). Medical students' preparedness for professional activities in early clerkships. BMC Med Educ.

[12] Rasch G (1980). Studies in Mathematical Psychology I: Probabilistic Models for Some Intelligence and Attainment Tests.

[13] Tesio L, Caronni A, Simone A, Kumbhare D, Scarano S (2024). Interpreting results from Rasch analysis 2. Advanced model applications and the data-model fit assessment. Disabil Rehabil.

[14] Hope D, Kluth D, Homer M, Dewar A, Goddard-Fuller R, Jaap A, Cameron H (2025). Exploring the use of Rasch modelling in "common content" items for multi-site and multi-year assessment. Adv Health Sci Educ Theory Pract.

[15] Tavakol M, Pinner G (2019). Using the many-facet Rasch model to analyse and evaluate the quality of objective structured clinical examination: a non-experimental cross-sectional design. BMJ Open.

[16] Tor E, Steketee C (2011). Rasch analysis on OSCE data : an illustrative example. Australas Med J.

[17] Aryadoust V, Ng LY, Sayama H (2020). A comprehensive review of Rasch measurement in language assessment: recommendations and guidelines for research. Lang Test.

[18] Hanaki T (2025). Step Ladder System (SLS) Task List (ver. 1.7) from the Division of Gastrointestinal and Pediatric Surgery, Tottori University Faculty of Medicine. figshare.

[19] Tomczak M, Tomczak-Łukaszewska E (2014). The need to report effect size estimates revisited. An overview of some recommended measures of effect size. Trends Sport Sci.

[20] Bond T, Yan Z, Heene M (2020). Applying the Rasch Model. Applying the Rasch Model.

[21] Wright BD, Masters GN (1982). Rating scale analysis. https://www.rasch.org/BTD_RSA/pdf%20[reduced%20size]/Rating%20Scale%20Analysis.pdf.

[22] Guilleux A, Blanchin M, Hardouin JB, Sébille V (2014). Power and sample size determination in the Rasch model: evaluation of the robustness of a numerical method to non-normality of the latent trait. PLoS One.

[23] Linacre J (1994). Sample size and item calibration [or person measure] stability. Rasch Measurement Transactions.

